# Indirect reciprocity can foster large-scale cooperation

**DOI:** 10.1073/pnas.2409894121

**Published:** 2024-06-24

**Authors:** Jörg Gross, Zsombor Z. Méder, Angelo Romano, Carsten K. W. De Dreu

**Affiliations:** ^a^Department of Psychology, University of Zurich, Zurich 8050, Switzerland; ^b^Faculty of Economics and Business, Groningen University, Groningen 9700AB, the Netherlands; ^c^Department of Psychology, Leiden University, Leiden 2333AK, the Netherlands; ^d^Faculty of Behavioral and Social Sciences, University of Groningen, Groningen 9712TS, the Netherlands; ^e^Behavioral Ecology and Sociobiology Unit, German Primate Center, Leibniz Institute for Primate Research, Göttingen 37077, Germany

Schnell and Muthukrishna (1) (S&M) present an intriguing theoretical model, extending indirect reciprocity to group-structured populations. In their model, agents interact across two stages. First, each agent chooses one of the following actions: defect, cooperate only with members of their own group, or cooperate with members of other groups. Second, in a “Mutual Aid Game” (MAG), agents are paired with a partner and can create a benefit for them conditional on the target’s reputation. The authors conclude that under a range of reputation rules, indirect reciprocity alone “is insufficient for stabilizing large-scale human cooperation.”

The model makes one important assumption: Agents only interact with fellow in-group members in the MAG. This raises three important questions: a) Can indirect reciprocity, in theory, promote large-scale cooperation when allowing for interactions across group boundaries such that reputation toward out-group members is also pertinent? b) Do people actually implement conditional reputation strategies when interacting with out-group members? c) To which degree does belonging to a group restrict interaction frequency with out-group members in humans and other animals?

Based on research on local vs. global cooperation (2–4) new results of a more general theoretical model (5), also quoted by S&M, show that when interactions across group boundaries become possible, an even simpler reciprocity mechanism is sufficient to promote large-scale cooperation. The underlying idea is the following: With frequent intergroup interactions, agents should not only consider how in-group members judge and act in the MAG but also how out-group members react to cooperative actions. With sufficient intergroup interactions, strategies can evolve that help those who cooperate across group boundaries, paving the way for large-scale cooperation (5).

But do people actually enforce intergroup cooperation when meeting out-group members? In an experiment, participants played a game similar to the one studied by S&M, but they frequently interacted with members of different groups in the MAG. This showed that a) people provide aid to out-group members and in-group members alike (Fig. 1*A*), and b) they do so conditionally (Fig. 1*B*). Similar to the strategies analyzed by S&M, people provided more aid to others who also provided aid to cooperators rather than defectors. Importantly, out-group members rewarded intergroup cooperators more strongly than in-group cooperators, thereby fostering large-scale cooperation. Thus, groups can mutually reinforce intergroup cooperation similar to how individuals can mutually reinforce dyadic cooperation through indirect reciprocity.

**Fig. 1.**
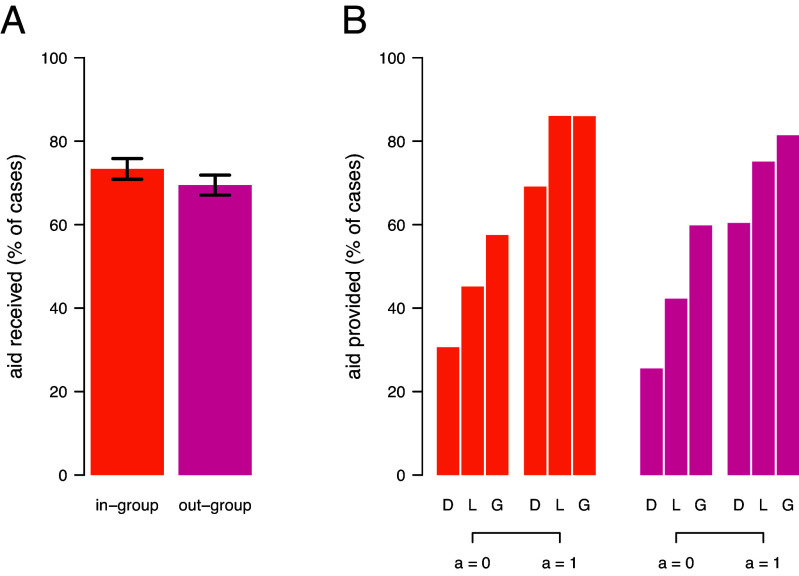
(*A*) Average aid received in the Mutual Aid Game (MAG) from in-group members (yellow) or out-group members (magenta). (*B*) Aid provided to other participants depending on whether the target provided help in the previous MAG (a = 1) or not (a = 0) and whether the target did not cooperate (D; “defector”), contributed to the local PGG (L; “in-group cooperator”), or the global PGG (G; “intergroup cooperator”), separated by meeting in-group members (yellow) or out-group members (magenta) in the MAG. Data analyzed from the “fluid boundary condition” in ref. 5.

This suggests that indirect reciprocity can foster large-scale cooperation and that individuals use the MAG to shift others’ behavior from in-group to intergroup cooperation. This leaves our third question—does belonging to a group in itself prevent interactions with out-group members? The answer is negative. Cross-group tolerance and cooperative exchange are seen across a range of group-living species, from social insects (6) to both nonhuman and human primates (7, 8). More importantly, how frequent are such interactions crossing group boundaries? This likely depends on the underlying socioecology (9, 10). For example, with larger group sizes and growing social mobility, the likelihood of meeting individuals from out-groups increases (5, 10). Hence, fitting (2–10), when interactions transcend group boundaries, indirect reciprocity can enable the evolution of large-scale cooperation.

## References

[1] E. Schnell, M. Muthukrishna, Indirect reciprocity undermines indirect reciprocity destabilizing large-scale cooperation. Proc. Natl. Acad. Sci. U.S.A. **121**, e2322072121 (2024). 38683991 10.1073/pnas.2322072121 PMC11087788

[2] T. Yamagishi, T. Kiyonari, The group as the container of generalized reciprocity. Soc. Psychol. Q. **63**, 116 (2000).

[3] B. Simpson, B. Montgomery, D. Melamed, Reputations for treatment of outgroup members can prevent the emergence of political segregation in cooperative networks. Nat. Commun. **14**, 7721 (2023). 38001105 10.1038/s41467-023-43486-7 PMC10674010

[4] A. Romano, D. Balliet, T. Yamagishi, J. H. Liu, Parochial trust and cooperation across 17 societies. Proc. Natl. Acad. Sci. U.S.A. **114**, 12702–12707 (2017). 29133403 10.1073/pnas.1712921114 PMC5715771

[5] J. Gross , The evolution of universal cooperation. Sci. Adv. **9**, eadd8289 (2023). 36800427 10.1126/sciadv.add8289 PMC9937576

[6] A. Rodrigues, J. Barker, E. Robinson, From inter-group conflict to inter-group cooperation: Insights from social insects. Philos. Trans. R Soc. B Biol. Sci. **377**, 20210466 (2022). 10.1098/rstb.2021.0466 PMC8977659 35369743

[7] A. C. Pisor, M. Surbeck, The evolution of intergroup tolerance in nonhuman primates and humans. Evol. Anthropol. Issues News Rev. **28**, 210–223 (2019). 10.1002/evan.21793 31386248

[8] L. Samuni, M. Surbeck, Cooperation across social borders in bonobos. Science **382**, 805–809 (2023). 37972165 10.1126/science.adg0844

[9] C. K. W. De Dreu, J. Gross, A. Romano, Group formation and the evolution of human social organization. Perspect. Psychol. Sci. **19**, 324–334 (2023). 10.1177/17456916231179156 PMC10913362 37450408

[10] R. Thomson , Relational mobility predicts social behaviors in 39 countries and is tied to historical farming and threat. Proc. Natl. Acad. Sci. U.S.A. **115**, 7521–7526 (2018). 29959208 10.1073/pnas.1713191115 PMC6055178

